# Genotype and phenotype correlations in Iranian patients with hyperinsulinaemic hypoglycaemia

**DOI:** 10.1186/s13104-015-1319-1

**Published:** 2015-08-13

**Authors:** Senthil Senniappan, Atefeh Sadeghizadeh, Sarah E Flanagan, Sian Ellard, Mahin Hashemipour, Majid Hosseinzadeh, Mansour Salehi, Khalid Hussain

**Affiliations:** Alder Hey Children’s Hospital, Liverpool, UK; Department of Pediatrics, Child Growth and Development Research Center, Isfahan University of Medical Sciences, Isfahan, Iran; Institute Biomedical and Clinical Science, University of Exeter Medical School, Exeter, EX2 5DW UK; Endocrinology and Metabolism Research Center, Isfahan University of Medical Sciences, Isfahan, Iran; Medical Genetics Laboratory, Alzahra University Hospital, Isfahan University of Medical Sciences, Isfahan, Iran; Pediatric Inherited Disease Research Center (PIDRC), Isfahan University of Medical Sciences, Isfahan, Iran; Developmental Endocrinology Research Group, Clinical and Molecular Genetics Unit, Institute of Child Health, University College London, London, UK; Department of Paediatric Endocrinology, Great Ormond Street Hospital for Children, London, UK

**Keywords:** Hyperinsulinaemic hypoglycaemia, HADH (hydroxyacyl-CoA-dehydrogenase), Diazoxide

## Abstract

**Background:**

Hyperinsulinaemic hypoglycaemia (HH) is a group of clinically and genetically heterogeneous disorders characterized by unregulated insulin secretion. Abnormalities in nine different genes (*ABCC8, KCNJ11, GLUD1, GCK, HADH, SLC16A1, HNF4A, UCP2 and HNF1A*) have been reported in HH, the most common being *ABCC8* and *KCNJ11*. We describe the genetic aetiology and phenotype of Iranian patients with HH.

**Methods:**

Retrospective clinical, biochemical and genetic information was collected on 23 patients with biochemically confirmed HH. Mutation analysis was carried out for the ATP-sensitive potassium (K_ATP_) channel genes (*ABCC8 and KCNJ11*), *GLUD1, GCK, HADH* and *HNF4A*.

**Results:**

78 % of the patients were identified to have a genetic cause for HH. 48 % of patients had mutation in *HADH,* whilst *ABCC8/KCNJ11* mutations were identified in 30 % of patients. Among the diazoxide-responsive patients (18/23), mutations were identified in 72 %. These include two novel homozygous *ABCC8* mutations. Of the five patients with diazoxide-unresponsive HH, three had homozygous *ABCC8* mutation, one had heterozygous *ABCC8* mutation inherited from an unaffected father and one had homozygous *KCNJ11* mutation. 52 % of children in our cohort were born to consanguineous parents. Patients with *ABCC8/KCNJ11* mutations were noted to be significantly heavier than those with *HADH* mutation (p = 0.002). Our results revealed neurodevelopmental deficits in 30 % and epilepsy in 52 % of all patients.

**Conclusions:**

To the best of our knowledge, this is the first study of its kind in Iran. We found disease-causing mutations in 78 % of HH patients. The predominance of *HADH* mutation might be due to a high incidence of consanguineous marriage in this population. Further research involving a larger cohort of HH patients is required in Iranian population.

## Background

Hyperinsulinaemic hypoglycaemia (HH) is a group of clinically and genetically heterogeneous disorders characterized by dysregulation of insulin secretion by pancreatic β-cells [1]. Early diagnosis and treatment is important to prevent permanent brain damage [2]. HH can be either transient or persistent; transient forms of HH are usually secondary to conditions such as maternal diabetes mellitus or intra-uterine growth retardation [1]. The incidence of HH can vary from 1 in 35,000–40,000 in the general population [3] to 1 in 2,500 in some communities with high rates of consanguinity [4].

The clinical presentation can be varied ranging from completely asymptomatic, mild disease to severe disease unresponsive to medication needing surgical intervention [5]. The HH due to recessive mutations in *ABCC8/KCNJ11* is usually severe and requires high concentrations of intravenous glucose to maintain normoglycaemia [5]. Hypoglycemic symptoms may vary from being non-specific (such as poor feeding, lethargy and irritability) to severe (such as apnea, seizures or coma). Macrosomia is a common feature in infants, but not all babies with HH are macrosomic [6]. There are two histological subtypes of CHI: diffuse and focal [7]. The diffuse form is inherited in an autosomal recessive (or dominant) manner whereas the focal form is sporadic in inheritance. The first line of medical therapy in HH includes diazoxide, which binds to the intact SUR1 component of the K_ATP_ channels and prevents depolarization of the β-cell membrane and insulin secretion [1].

HH is caused by mutations in the key genes that are involved in regulation of insulin secretion from the pancreatic β-cells. So far, mutations in *ABCC8, KCNJ11, GLUD1, GCK, HADH, SLC16A1, HNF4A, UCP2* and *HNF1A* have been identified to be involved in the pathogenesis of HH [1, 8]. The most common causes of diffuse medically unresponsive HH are mutations in *ABCC8* and *KCNJ11*. These two genes encode for the SUR1 (sulphonylurea receptor 1 subunit) and Kir6.2 (inward-rectifying potassium channel pore-forming subunit) proteins, respectively which constitute the K_ATP_ channel of the pancreatic β-cell membrane [2].

The inactivating mutations in *ABCC8/KCNJ11* reduce or completely abolish the activity of the K_ATP_ channel, leading to unregulated insulin release despite severe hypoglycaemia [9]. The recessive inactivating mutations in *ABCC8* and *KCNJ11* usually cause severe HH, which is unresponsive to medical treatment with diazoxide. The molecular basis of recessive inactivating *ABCC8* and *KCNJ11* mutations involve defects in K_ATP_ channel biogenesis and turnover, channel trafficking from the ER and Golgi apparatus to the plasma membrane and alterations of channels in response to nucleotide regulation and open state frequency [10]. Dominant inactivating mutations in *ABCC8* and *KCNJ11* usually cause HH with a milder phenotype [11].

Hyperinsulinism–hyperammonaemia syndrome (HI/HA), the second most common form of HH is associated with activating missense mutations in *GLUD1*, which encodes the mitochondrial matrix enzyme, glutamate dehydrogenase (GDH). Patients present with recurrent symptomatic postprandial hypoglycaemia following protein-rich meals (leucine-sensitive hypoglycaemia) as well as fasting hypoglycaemia accompanied by asymptomatic elevations of plasma ammonia [12]. Mutations in *HNF4A, HNF1A* and *GCK* cause maturity-onset diabetes of the young (MODY) as well HH [8, 13].

Mutations in the mitochondrial *HADH* gene (encoding the enzyme L-3-hydroxyacyl-coenzyme A dehydrogenase, HADH), are a rare cause of HH [14]. This enzyme catalysis the conversion of L3-hydroxyacyl-CoAs of variable chain length to their corresponding 3-ketoacyl-CoAs and exerts highest activity to 3-hydroxybutyryl-CoA. *HADH* mutations can lead either to severe neonatal HH or to mild late onset HH [15]. All patients reported so far have responded to diazoxide and some had abnormal acylcarnitine metabolites (raised plasma hydroxybutyrylcarnitine and urinary 3-hydroxyglutarate levels). Protein sensitivity has been demonstrated in patients with HADH mutations [16] and this has been confirmed in the HADH knockout mouse [17]. However the precise mechanism of dysregulated insulin secretion in patients with a HADH deficiency is not understood but might involve an interaction between GDH and HADH [17]. Genetic analysis for HADH gene is recommended in patients with diazoxide responsive HH from consanguineous families, who are negative for mutations in the K_ATP_ channels [18].

Although clinical characteristics and genetic etiology of HH patients have been described in some studies [19–21], little is known about HH in the Iranian population with a high rate of consanguineous marriages [22]. The aim of this study was to investigate genotype/phenotype correlations in a sample of Iranian patients with HH from Isfahan.

## Methods

In this cross-sectional study, we collected data on the patients who visited the pediatric endocrinology outpatient clinics and the infants who were referred to Al-Zahra Hospital from September 1998 to July 2012. All infants and children were diagnosed with HH based on clinical and biochemical criteria [2]. Patients with a secondary cause of HH such as perinatal asphyxia, prematurity, intra-uterine growth restriction, and syndromic forms were excluded. The clinical data included age at presentation, birth weight, medications, neonatal history, epilepsy and neurological deficits, family history, history of hypoglycaemia following protein-rich meals and consanguinity of parents. Serum ammonia level was checked in all patients.

Children were defined as being responsive to diazoxide when (a) feeding with normal frequency and volume (b) able to fast appropriately for age and maintain normal blood glucose levels (c) serum insulin level low or undetectable at the end of the fast (d) appropriate increase in serum fatty acids and ketone bodies at the end of the fast [2]. Diazoxide-unresponsive patients either underwent near total pancreatectomy or were managed with combinations of octreotide and diazoxide along with high calorie feeds.

Written informed consent for genetic tests was obtained from parents of all patients. The study was reviewed and approved by the Ethical Committee of Isfahan University of Medical Sciences.

Genomic DNA was extracted from peripheral leukocytes using standard procedures. All exons and intron–exon boundaries of *ABCC8, KCNJ11, HADH, GLUD1, HNF4A* and *GCK* genes were amplified by PCR. The products were sequenced using a BigDye Terminator v3.1 Cycle sequencing Kit on an ABI 3730XL Analyzer (Applied Biosystems, Foster City, CA, USA) and sequences were compared with the reference sequence (NM_000525 for KCNJ11, NM_000352.3 for ABCC8, and NM_000162.3 for GCK) using Chromas (V.2.01, Technelysium Pty Ltd, Tewantin QLD, Australia) or Mutation Surveyor software V3.24 (Softgenetics, State College, PA, USA). Mutation testing was done on parental samples when a mutation was identified in the child. If no mutation was identified, testing for a partial/whole gene deletion was undertaken using multiplex ligation-dependent probe amplification (MLPA).

### Statistical analysis

Data are presented as mean ± standard deviation and frequency. The independent sample t test was used to assess continuous variables between two groups. The Mann–Whitney test was used for data that were not normally distributed. Analyses were performed using SPSS (IL, USA, version 19.0). Significant level was set as P < 0.05.

## Results

Twenty-three patients with HH with age ranging from 1 month to 21 years were included in this study. One patient with achondroplasia and three children with secondary HH due to intra-uterine growth restriction were excluded. The age at presentation ranged from neonatal period (day 1 of life) to 3 years. Eleven (47 %) of the patients were noted to be symptomatic within the first 48 h after birth. The most common clinical symptom was seizure (82 %). All patients except two children were born at full term. The mean birth weight was 3,423 ± 757 g (with a range of 1,700–5,000 g). Only five patients (21 %) were macrosomic. Ten (43 %) of patients were female. 12 (52 %) children with HH were born to consanguineous couples. Two families had two affected children.

A high frequency (30 %) of neurodevelopmental delay was observed in these patients. Furthermore, 12 (52 %) patients suffered from epilepsy. None of the patients had hyperammonemia.

A total number of 18 (78 %) patients responded to diazoxide. Five diazoxide-unresponsive patients underwent near total pancreatectomy. One of them died at 4 months of age due to thromboembolism in the post-operative period. One other patient, who was managed with combinations of octreotide and diazoxide, died at 3 months of age due to sepsis. Clinical characteristics and gene mutations of these patients were summarized in Table 1.

**Table 1:** Clinical characteristics and genetic mutations of 23 Iranian patients with hyperinsulinaemic hypoglycaemia

Patient number	Gender (F = female, M = male)	Gestation-term	Birth weight (g)	Age of onset of hypoglycaemia	Epilepsy	Neurodevelopmental delay	Consanguineous parents	Treatment			Genotype	Mutation	Maternal/paternal genotype	Follow up
								Diazoxide responsive	Octreotide responsive	Pancreatectomy				
*ABCC8*														
1	F	Yes	3,280	2 days	No	No	No	Yes	Yes		Heterozygous	c.2041-21G>A (splicing)	None/heterozygous	Died at 3 months of ago
2	F	Yes	3,750	1 day	Yes	Yes	No			Yes	Homozygous	p.R1494W (missense)	Heterozygous/heterozygous	Needed insulin
3	F	Yes	4,200	1 day	Yes	No	Yes			Yes	Homozygous	p.W143X (nonsense)	Heterozygous/heterozygous	Needed diazoxide
4	F	Yes	5,000	1 day	No	No	Yes			Yes	Homozygous	p.G1376R (missense)	Heterozygous/heterozygous	Needed diazoxide
5	M	Yes	4,200	1 day	No	No	Yes	Yes			Homozygous	c.2697+5G>A (intron 22, novel splicing)	Heterozygous/heterozygous	On diazoxide
6	M	Yes	4,500	1 day	No	No	Yes	Yes			Homozygous	c.2697+5G>A (intron 22, novel splicing)	Heterozygous/heterozygous	Resolved by 4 years of ago
*KCNJ11*														
7	M	Yes	4,400	1 day	No	No	Yes			Yes	Compound heterozygous	p.P34OH (missense) and pF117del (novel in-frame)	Heterozygous/heterozygous	Died at 4 months of ago
*HADH*														
8	M	Yes	2,860	3 months	Yes	No	Yes	Yes			Homozygous	delA617.c (frameshift)	Heterozygous/heterozygous	On diazoxide
9	F	Yes	3,000	1 year	No	No	Yes	Yes			Homozygous	delA617.c (frameshift)	Heterozygous/heterozygous	Stopped diazoxide/no relapse
10	M	Yes	2,900	1 day	Yes	Yes	Yes	Yes			Homozygous	delA617.c (frameshift)	Heterozygous/heterozygous	On diazoxide
11	F	Yes	3,650	4 days	No	No	No	Yes			Homozygous	delA617.c (frameshift)	Heterozygous/heterozygous	On diazoxide
12	M	Yes	3,600	3 months	Yes	Yes	Yes	Yes			Homozygous	delA617.c (frameshift)	Heterozygous/heterozygous	On diazoxide
13	M	Yes	3,100	1.5 months	Yes	Yes	No	Yes			Homozygous	delA617.c (frameshift)	Heterozygous/heterozygous	On diazoxide
14	M	Yes	2,900	7.5 months	No	No	Yes	Yes			Homozygous	delA617.c (frameshift)	Heterozygous/heterozygous	On diazoxide
15	F	Yes	3,200	1 day	Yes	No	No	Yes			Homozygous	delA617.c (frameshift)	Heterozygous/heterozygous	On diazoxide
16	M	Yes	3,600	3 months	Yes	Yes	No	Yes			Homozygous	delA617.c (frameshift)	Heterozygous/heterozygous	On diazoxide
17	M	Yes	3,500	3 months	Yes	Yes	No	Yes			Homozygous	delA617.c (frameshift)	Heterozygous/heterozygous	On diazoxide
18	M	Yes	3,500	3 months	Yes	No	No	Yes			Homozygous	delA617.c (frameshift)	Heterozygous/heterozygous	On diazoxide
No mutation														
19	M	No	1,700	3 years	No	No	Yes	Yes						Tapered diazoxide/no relapse
20	M	No	2,050	1 day	Yes	No	No	Yes						On diazoxide
21	F	Yes	2,800	9 months	No	No	No	Yes						On diazoxide
22	F	Yes	3,300	5 months	Yes	Yes	Yes	Yes						On diazoxide
	F	Yes	3,750	1 day	No	No	No	Yes						On diazoxide

**Fig. 1:**
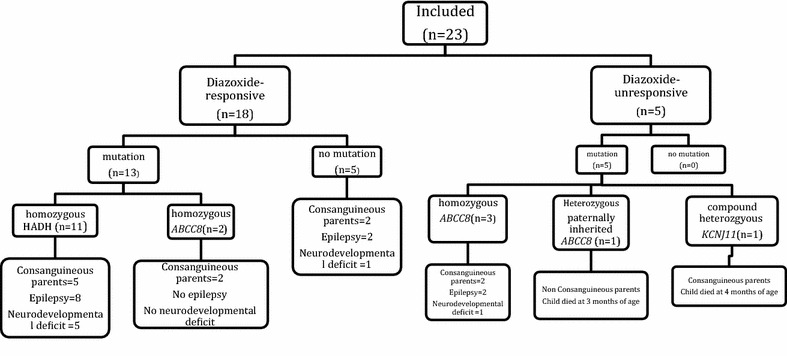
Summary of the genotype–phenotype correlation and long term outcome.

Disease causing mutations were identified in 78 % of the patients (48 % had *HADH* mutation, 26 % had *ABCC8* and 4 % had *KCNJ11* mutation) (Fig. 1). Of the five with diazoxide-unresponsive HH, three had homozygous *ABCC8* mutation, one had heterozygous *ABCC8* mutation, which was inherited from an unaffected father, and one had homozygous *KCNJ11* mutation. All three diazoxide unresponsive patients with homozygous *ABCC8* mutation were managed by subtotal pancreatectomy. One of these patients needed insulin due to hyperglycemia in the post-operative period. The other two patients were euglycemic on a small (5 mg/kg/day) dose of diazoxide. One patient with a paternally inherited mutation was treated non-surgically with octreotide and diazoxide. One patient with homozygous *KCNJ11* mutation was managed by total pancreatectomy.

Interestingly two patients with a novel homozygous intronic *ABCC8* mutation were diazoxide responsive and HH resolved by 4 years of age in one of the two patients. The mechanism of this is not clear.

The same homozygous *HADH* mutation was identified in 11 children with diazoxide-responsive HH [frameshift mutation c.617delA, which is a deletion of an A nucleotide at position 617 and is predicted to result in a premature termination at codon 219 (p.K206fsX14)]. This included two affected siblings in two different families. Eight (72 %) children with *HADH* mutation had a history of hypoglycemia following protein-rich meals, although no formal protein load test was done in these patients.

Infants with *ABCC8*/*KCNJ11* mutations were heavier at birth in comparison to the patients with *HADH* mutation (4,190 ± 550 g vs. 3,255 ± 318 g, P = 0.002). The mean age of presentation of patients with a *HADH* mutation was 99.1 days in comparison to 1.14 days in patients with *ABCC8* mutations; the difference was statistically significant (P = 0.004).

## Discussion

In this study, 78 % of patients with HH from Isfahan, Iran were noted to have disease-causing mutations. It is very interesting to note that *HADH* mutation was the commonest genetic cause in this cohort (48 %) as opposed to the *ABCC8* mutations, which was noted only in 26 % of patients. The higher incidence of patients with *HADH* mutation accounted for the higher rate of diazoxide responsiveness in this group (78 %). All diazoxide-unresponsive patients in this series had identifiable mutations (*ABCC8/KCNJ11*), whilst no mutation was identified in 28 % of diazoxide-responsive patients.

In contrast to these observations, several other studies suggest a major role of K_ATP_ channel in the pathogenesis of patients with HH [19, 21, 23]. In a large series of 417 patients with HH, mutations were identified in 91 % of diazoxide-unresponsive probands, and in 47 % of diazoxide-responsive patients [20]. However, only 2 cases (4 % of diazoxide-responsive probands) were positive for *HADH* mutation. In another large series involving 300 patients with HH [19], mutations were identified in 45.3 % of patients and *ABCC8/KCNJ11* mutations were noted to be the commonest. Among the 22.4 % of patients who had mutations in the diazoxide responsive group, only 3 patients (1 % of all cases) had *HADH* mutation [19].

In keeping with previous observation [14, 19, 20], all patients with *HADH* mutation in this group were diazoxide responsive, whilst the majority of patients (71 %) with *ABCC8/KCNJ11* mutations were diazoxide-unresponsive. As shown previously, patients with *HADH* mutation were diagnosed later and were of normal birth weight in comparison to *ABCC8* mutations [19, 24].

The clinical presentation of patients with heterozygous (autosomal dominant) *ABCC8* mutations is variable, ranging from mild medically responsive forms to severe early-onset HH and the vast majority of patients who do not respond to diazoxide have homozygous *ABCC8* mutations [20]. We observed that three of the five patients with severe HH had homozygous *ABCC8* mutations. Interestingly a novel homozygous *ABCC8* mutation was identified in two patients with a mild form of HH that resolved completely. The precise mechanism of the course of HH in these patients is unclear.

Genetic analysis of *HADH* is generally suggested in patients with diazoxide responsive HH from consanguineous families, who are negative for mutations in the K_ATP_ channels [18]. The Iranian population like some other populations, has a high level of consanguinity [22]. A higher rate of consanguineous marriages may favor the onset and increased frequency of autosomal recessive diseases in a population [22]. Parental consanguinity was observed in 45 % (5/11) of patients with HADH mutation in our cohort. Hence the higher incidence of HADH mutation in our group of patients is very likely related to the higher rate of consanguineous marriages in these families. Also 80 % (4/5) of children with homozygous *ABCC8* mutation had consanguineous parents.

Our results showed neurodevelopmental deficits in 30 % of the patients and epilepsy in 52 % of all cases. The prevalence of mental retardation and epilepsy was reported to be 31 and 15 %, respectively in a group of patients with HH in Austria [25]. Long-term follow up of 114 patients with HH showed poor general outcome with a high degree of psychomotor or mental retardation (44 %) or epilepsy (25 %) [26]. Another study from Argentina reported neurological impairment in 38 % of children with HH [27], whereas a study from Greece which followed up 13 HH patients reported a good neurological outcome without any psychomotor retardation [28]. It is well known that neonatal hypoglycemia is associated with neurodevelopmental complications [29–31].

Our study has several limitations. Firstly it includes a small number of patients from Isfahan, which may not be representative of the whole Iranian population. Secondly the data was collected on a retrospective basis from the medical records. Finally, an 18-F DOPA PET/CT scan was not available to differentiate focal from diffuse lesion.

## Conclusion

This study first of its kind from Iran, demonstrates disease-causing mutations in 78 % of HH patients from Isfahan. Majority of these patients (78 %) responded to medical therapy with diazoxide. A high rate (48 %) of *HADH* mutation is seen in this population that might be attributed to a high rate of consanguineous marriages. Data shows a suboptimal long-term outcome with a high degree of neurodevelopmental deficits and epilepsy in these patients. Further research with a larger number of patients is necessary to identify the mechanism(s) of HH in Iranian population.

## Abbreviations

GCK: glucokinase
GDH: glutamate dehydrogenase
HI/HA: hyperinsulinism–hyperammonaemia syndrome
HH: hyperinsulinaemic hypoglycaemia
HADH: hydroxyacyl-coenzyme A dehydrogenase
HNF: hepatocyte nuclear factor
MLPA: multiplex ligation-dependent probe amplification
MODY: maturity onset diabetes of the young
PCR: polymerase chain reaction
K_ATP_: potassium ATP channel
SUR: sulphonylurea receptor
